# Supplementary figures and images for: Comprehensive Transcriptome Analysis of Follicles from Two Stages of the Estrus Cycle of Two Breeds Reveals the Roles of Long Intergenic Non-Coding RNAs in Gilts
Source: Biology (Basel). 2022 May 6;11(5):716. doi: 10.3390/biology11050716 (PMC9138455; doi:10.3390/biology11050716)

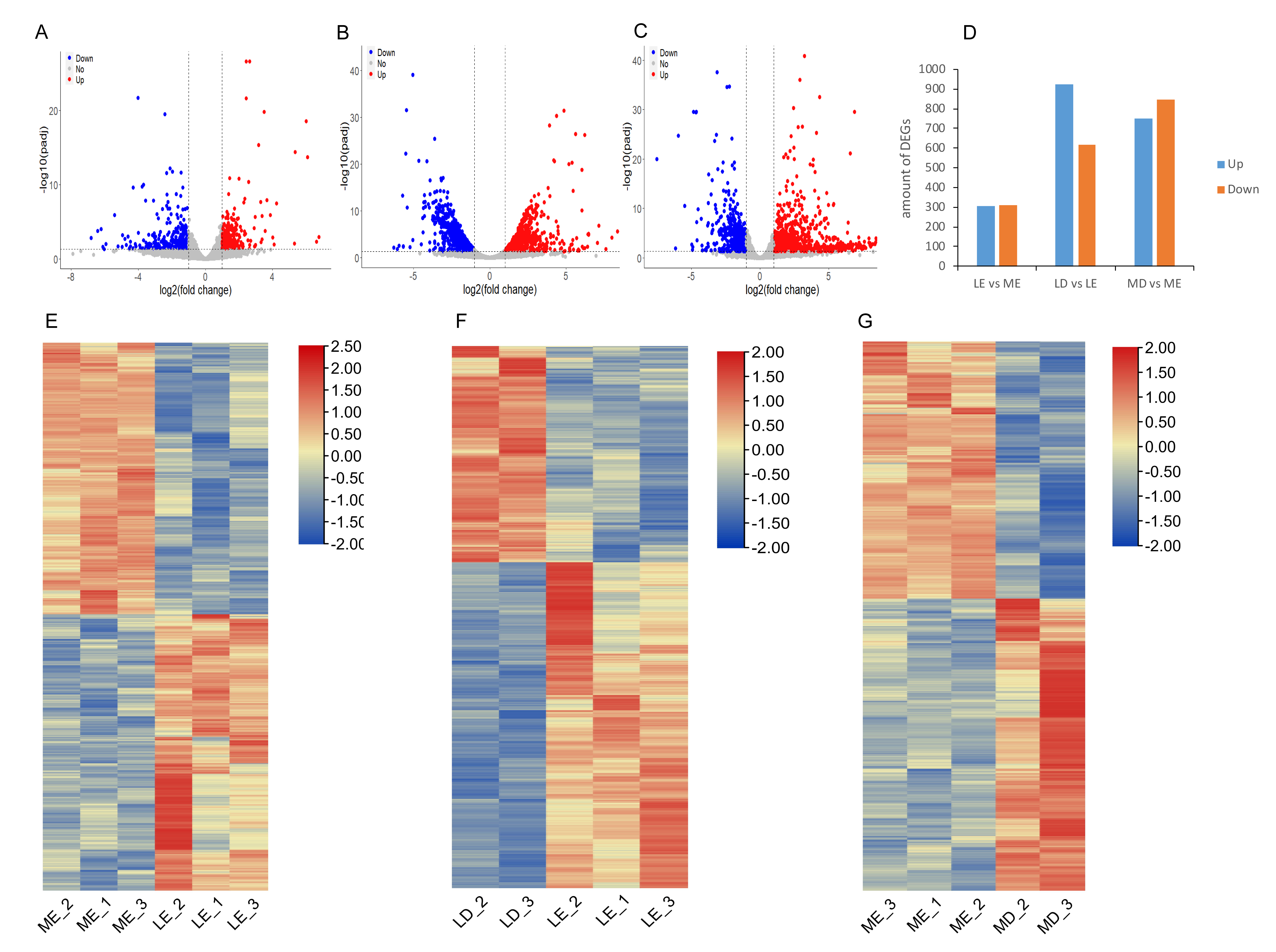

Supplement: Supplementary file 1 [file biology-11-00716-s001.zip › Supplementary files/Supplementary figure S1.tif]

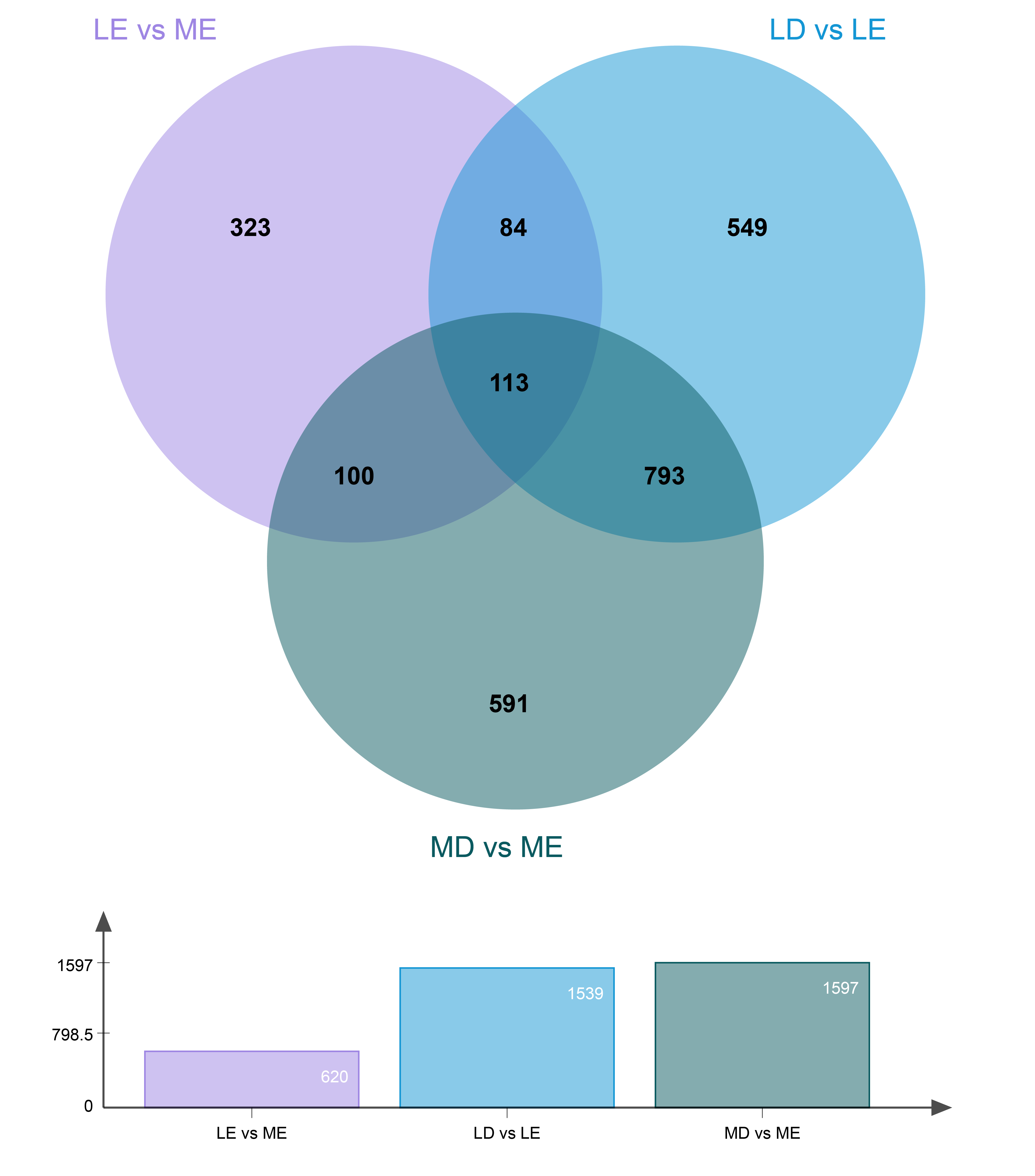

Supplement: Supplementary file 1 [file biology-11-00716-s001.zip › Supplementary files/Supplementary figure S2.tif]

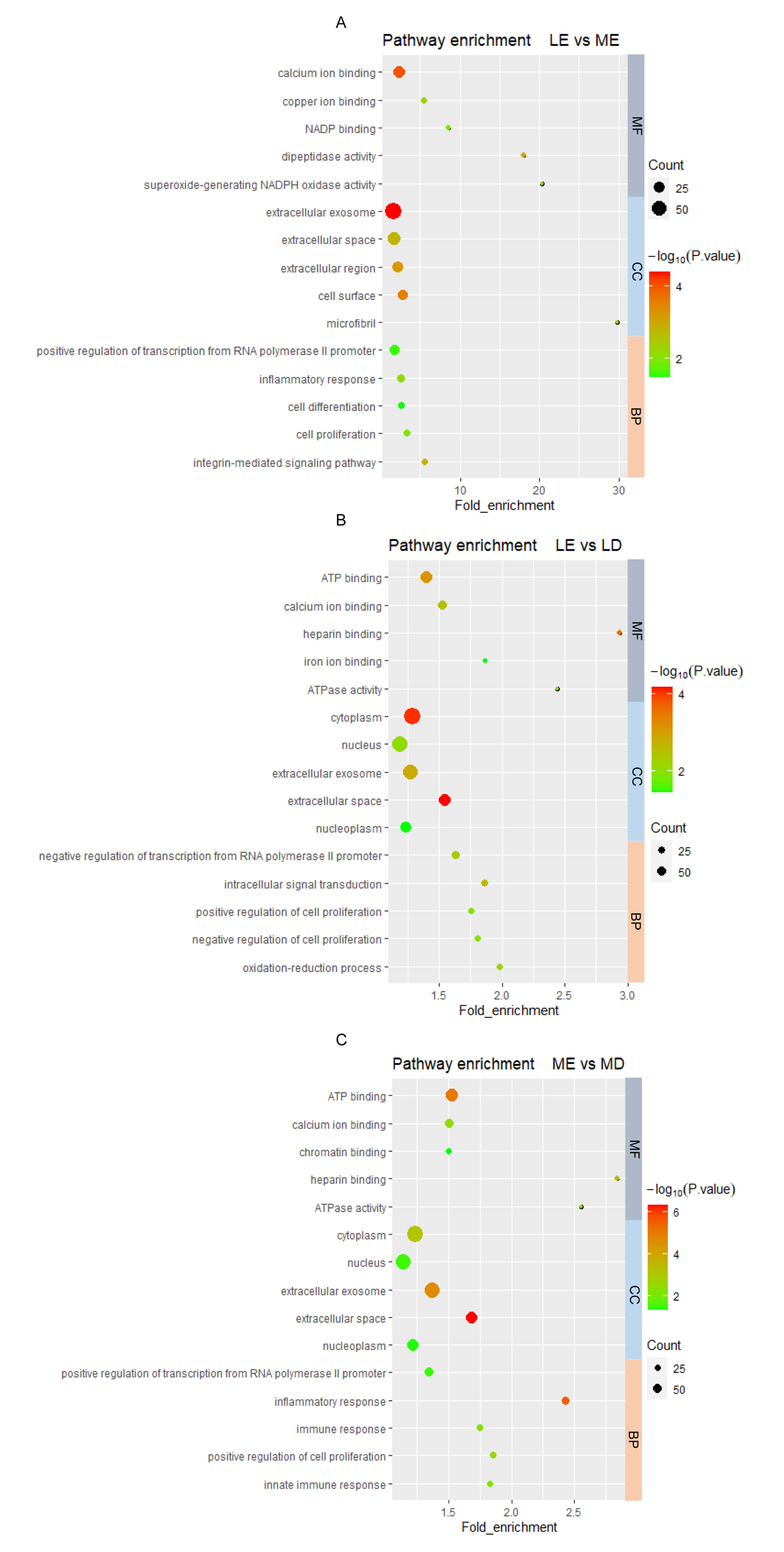

Supplement: Supplementary file 1 [file biology-11-00716-s001.zip › Supplementary files/Supplementary figure S3.tif]

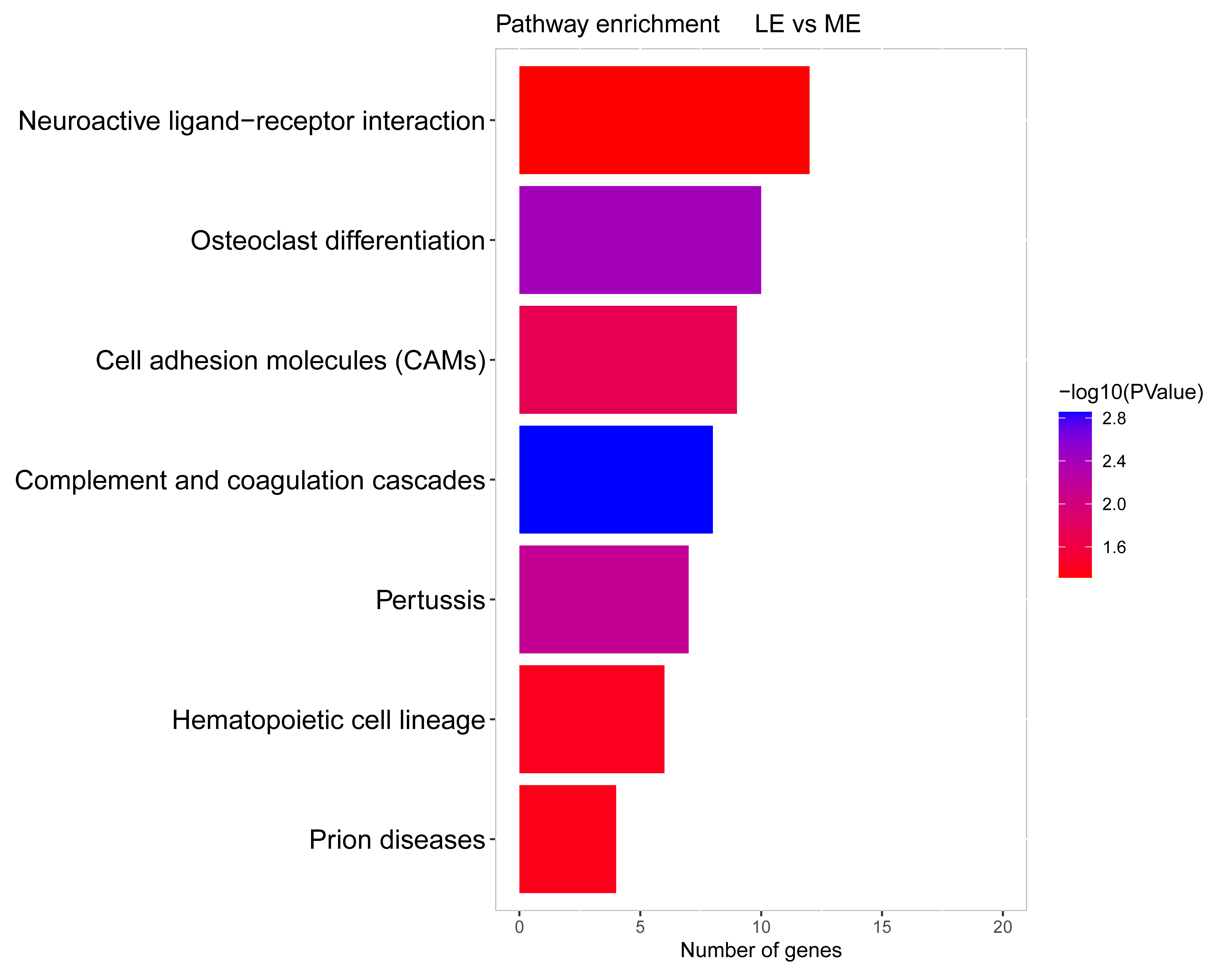

Supplement: Supplementary file 1 [file biology-11-00716-s001.zip › Supplementary files/Supplementary figure S4.tif]
